# Invasive group a streptococcal infection associated with community healthcare services delivered at home, South East England, December 2021–2023: Descriptive epidemiological study

**DOI:** 10.1017/S0950268825000287

**Published:** 2025-03-21

**Authors:** Jeeva John, Sonia Smith, Clare Sawyer, Beth Brokenshire, Charlotte Anderson, David J. Roberts

**Affiliations:** 1 UK Health Security Agency, South East, Chilton, Didcot, UK; 2 UK Health Security Agency, Field Service South East and London, London, UK

**Keywords:** community health services delivered at home, epidemiology, invasive Group A streptococcus, outbreak, Streptococcus pyogenes, wound infection

## Abstract

Invasive group A Streptococcal (iGAS) outbreaks have been linked to Community Healthcare Services Delivered at Home (CHSDH). There is, however, very limited evidence describing the epidemiology and mortality of iGAS cases associated with CHSDH. We used routine data to describe iGAS cases in adults who had received CHSDH prior to onset and compare characteristics between CHSDH-outbreak and non-outbreak CHSDH cases, in South East England between December 2021 and December 2023. There were 80/898 (8.9%) iGAS case episodes with CHSDH prior to onset; cases were in elderly people (50% aged 85 and over), and had primarily received wound or ulcer care (93.8%), with almost all care delivered by community nurses (98.8%). The 30-day all-cause case fatality was 26.3%. *Emm* 1.0 was the most common type (17.5%). In this period, 5/11 iGAS outbreaks (45.4%) were CHSDH-associated, and 25 cases with receipt of CHSDH prior to onset (31.3%, Confidence Interval [CI] 21.3–42.6%) were linked to these outbreaks. On univariate analysis, CHSDH-outbreak case episodes were more likely to be associated with *emm* pattern genotype E (OR 6.1 95% CI 1.8–20.9), and skin or soft tissue infection clinical presentation (OR 3.6, 95% CI 1.1–12.0) than non-outbreak CHSDH cases. There may be an increased risk of propagation of iGAS outbreaks in patients receiving CHSDH, emphasizing the need for rigorous early infection prevention and control, and outbreak surveillance.

## Background

Invasive group A Streptococcal (iGAS) bacterial infections are the most serious manifestations of a spectrum of diseases associated with GAS infection, with high case fatality [1]. Those aged over 75 years are at particular risk of severe disease [1, 2].

The risk of iGAS outbreaks in healthcare and institutional care settings is well described [3, 4], with surveillance studies finding that 10% of individual cases in healthcare settings were later associated with epidemiologically and molecularly defined clusters or outbreaks [4]. The risk of outbreaks associated with community healthcare is increasingly recognized; a 2021 Public Health England (PHE) review described an increase in iGAS outbreaks associated with Community Healthcare Services Delivered at Home (CHSDH), including in those receiving wound care [5]. Individuals receiving CHSDH may be at increased risk of iGAS infection, as this group often represents a particularly vulnerable cohort due to advanced age, and predisposing comorbidities, particularly skin breakdown [1, 5]. GAS transmission during healthcare may also be a particular risk in CHSDH, given difficulties implementing adequate Infection Prevention Control (IPC) within household settings [5], and transmission to patients with wounds or associated with wound care (a common CHSDH healthcare activity) is a recognized source of outbreaks in healthcare settings [4, 6] and in care homes [3]. Large droplet or direct contact transmission may propagate CHSDH-associated iGAS outbreaks via multiple routes from infected, colonized, or contaminated staff and patients, or by fomite transmission [5].

To our knowledge, no studies have described the detailed epidemiology and mortality of iGAS cases associated with CHSDH on a population basis, and the proportion of such iGAS cases that are part of a CHSDH-associated outbreak. This information may support risk assessment and case management, for which there is no guidance for this healthcare sector. We aimed to undertake a first regional, population-based description of iGAS cases and outbreaks associated with CHSDH, to inform decisions about further management and control of these infections, and surveillance and operational research.

## Methods

### Study design

This was a cross-sectional study using routinely collected data. We also did a case–case analysis, comparing iGAS cases linked to CHSDH-associated outbreaks to cases receiving CHSDH but not part of these outbreaks.

### Operational definitions

We included all confirmed and probable case episodes of iGAS in adults at least 18 years old and residents in South East England (Office for National Statistics 2022 mid-year adult population 7,416,948, of whom 922,111 were aged 75 years or older), notified to the South East Health Protection Team (HPT) between 1 December 2021 and 1 December 2023. Case definitions used were as outlined in public health guidance [2, 7]. Recurrent episodes were defined as occurring at least 28 days between episode onset dates. We measured case mortality from all causes within 30 days of the date of symptom onset. We defined iGAS outbreaks as at least two cases of confirmed or probable iGAS occurring within a 6-month period, with an epidemiological link by place or person (excluding those occurring exclusively within the same household), and linked by *emm* type and/or with supportive whole genome sequencing (WGS) Single Nucleotide Polymorphism (SNP) analysis (there being no defined SNP cutoff in guidance [2], and SNP variance being *emm* dependent [8], we checked outbreak/case notes did not report that the case(s) was/were subsequently excluded by the investigating outbreak control team (OCT) based on WGS SNP analyses). CHSDH-associated outbreaks were those where at least two cases were linked to the same CHSDH service without evidence of another more plausible route of transmission.

We defined CHSDH as community health services, including district nursing teams, general practitioners, podiatry (chiropody), community midwifery, hospital outreach, and palliative care, which provide medical or nursing care within a patient’s home or other ordinary residence, such as a care home. Exposure to CHSDH was defined as a case record indicating receipt of such care within 7 days prior to symptom onset, or within an unlimited time interval if the investigating HPT determined CHSDH as the most likely source of transmission based on epidemiological and molecular typing evidence within an outbreak investigation.

### Case management, outbreak detection and management, and microbiological characterization of iGAS isolates

Registered medical practitioners have a legal duty to notify suspected cases of iGAS disease, and diagnostic laboratories must report confirmed invasive *S. pyogenes.* Once notified, HPTs perform contact tracing and investigate potential sources of infection, including healthcare exposures. All iGAS sterile site isolates should be sent to the UKHSA reference laboratory for surveillance purposes, including for molecular typing of M protein (*emm*) gene. On receipt of typing, health protection staff investigated all notified iGAS cases for shared CHSDH services, alongside reviewing cases with shared *emm* type, to systematically detect clusters and potential outbreaks. On identifying a potential outbreak, the HPT can request WGS SNP analysis of isolates held by the reference laboratory to confirm or exclude epidemiologically linked cases of the same *emm* type from a cluster under investigation. The HPT would also conduct an outbreak investigation, supervized by an OCT undertaking epidemiological, microbiological, and environmental investigation, and review surveillance records to identify any common source or link between cases [2].

### Data sources

iGAS cases were identified from HPZone (InFact UK Ltd., Shipley, Yorkshire, BD17 7D, UK), the UKHSA Case and Incident Management System, and basic demographic and typing data were extracted. Each case was screened for CHSDH provision by searching for known flags from local case management protocols and by systematic keyword text searches within structured and unstructured casefile data. Following screening, we reviewed flagged case files to determine whether they met the operational definition of receiving CHSDH. Additionally, we reviewed all iGAS case records for all those aged 85-years-old and older.

For cases confirmed as having received CHSDH, we manually extracted further demographic, clinical, and exposure details and whether cases were part of an iGAS cluster or outbreak from HPZone case data, in agreement with a pre-specified protocol. Postcodes of case residence were used to allocate small-area level Index of Multiple Deprivation (IMD) 2019 quintiles as a measure of socioeconomic position. This was done using data held centrally by UKHSA, based on an open data source [9].

NHS numbers from HPZone records were used to link to supplementary data sources. Information on *emm* types (where missing from HPZone) was obtained from the UKHSA laboratory report database (Second Generation Surveillance System, SGSS); 30-day case mortality data was gathered from the NHS England Batch Demographics Service.

For iGAS outbreaks meeting the operational definition, we collected detailed epidemiological data from HPZone in agreement with a pre-specified protocol. For outbreaks that met the definition for being CHSDH associated, we contacted the OCT lead Consultant in Health Protection and/or Health Protection Practitioner to confirm that there was not another more plausible route of transmission.

### Statistical analysis

We used STATA (17.1, StataCorp LLC, College Station, TX) software for descriptive analysis of demographic, clinical, and microbiological characteristics of iGAS cases. We described case genetic subtype by *emm* type and *emm* pattern genotype, using a previously published classification [10]. We calculated the proportion of individual CHSDH-associated iGAS case episodes subsequently linked to a CHSDH iGAS outbreak for all CHSDH iGAS cases, and then a sensitivity analysis excluding cases with exposure >7 days prior to onset to quantify their impact on this proportion, as investigation of CHSDH exposure over this longer period was reserved for potential outbreak cases; their inclusion will therefore contribute disproportionately to the numerator. We used logistic regression to determine univariate associations of characteristics of CHSDH-exposed cases and characteristics of being associated with a CHSDH outbreak, estimating odds ratios (ORs) with 95% confidence intervals (CIs), with a sensitivity analysis excluding cases potentially recurring due to treatment failure.

### Ethical considerations

No ethical approvals were required as data used is routinely obtained and processed for communicable disease surveillance and control under section 251 of the National Health Service Act 2006.

## Results

There were 898 confirmed and/or probable iGAS case episodes notified in adults (312 in adults at least 75-years-old) between 1 December 2021 and 1 December 2023, giving an incidence of 6.05 and 16.92 per 100,000, respectively. Of these, (80/898 [8.9%]) case episodes (in 77 individuals) were recorded as having received CHSDH prior to symptom onset, increasing in frequency among those aged 85 years or older (40/127 [31.5%]).

Case episodes among people with CHSDH receipt are described in Table 1. These individuals were typically elderly (61/80 (76.3%) aged 75 and over), and nearly always received wound or ulcer care (75/80 [93.8%]), largely in their own home (72/80 [90.0%]), from community nurses (79/80 [98.8%]). CHSDH-associated iGAS case fatality within 30 days of onset was high (21/80 episodes [26.3%]). *Emm* 1.0 was the most commonly observed type (14/80 [17.5%]).

Among non-CHSDH-associated cases, individuals aged 75 years or older accounted for a smaller case percentage (251/818 [30.7%]), and in cases in this age group with typing available (204/251 [81.2%]) had a differing distribution of *emm* types (*emm* 1.0 101/204 [49.5%], *emm* 12.0 16/204 [7.8%], *emm* 89.0 13/204 [6.4%], *emm* 76.0 1/204 [0.5%]).

**Table 1.** Characteristics of iGAS case episodes in adults in receipt of CHSDH, South East England, December 2021–2023 (*n* = 80 episodes and *n* = 77 persons)

			Cases with CHSDH exposure	
			n	%
Demographics	Age, years	≤64 65–74 75–84 ≥85	10 9 21 40	12.5 11.3 26.3 50.0
	Sex	Female	42	52.5
	Type of home residence where CHSDH received	Care or residential home Own home	8 72	10.0 90.0
	Quintile of index of multiple deprivation	1 (least) 2 3 4 5 (most)	23 16 14 13 14	28.8 20.0 17.5 16.3 17.5
Exposure	Interval between CSHDH and onset	≤7 days >7 days	75 5	93.8 6.3
	CHSDH provider type	Community nursing only Mixed (Community nursing & other) Podiatry	76 3 1	95.0 3.8 1.3
	Type of care provided	Wound or ulcer care Other care Unknown	75 4 1	93.8 5.0 1.3
	Institutional care in preceding week	Yes No	6 74	7.5 92.5
	Symptomatic household contact	Yes No	7 73	8.8 91.3
Clinical outcomes	Mode of diagnosis	Blood culture Other sterile site isolate	79 1	98.8 1.3
	Clinical presentation	Any mention SSTI Other without SSTI Unknown	53 26 1	66.3 32.5 1.3
	30-day all-cause mortality	Dead Alive	21 59	26.3 73.8
	Recurrent episode	Yes	3	3.7
Microbiology	*Emm type*	1.0 76.0 89.0 12.0 Other Untypeable or unknown	14 11 9 7 34 5	17.5 13.8 11.3 8.8 42.5 6.3
	*Emm* pattern genotype	A–C (pharynx) D (skin) E (non-specific) Unknown	26 9 40 5	32.5 11.3 50.0 6.3

Abbreviation: SSTI, skin or soft tissue infection.

There were 11 iGAS outbreaks in the South East region during the 2-year analysis time-period; CHSDH was the most frequent shared exposure (5/11 (45.0%)), associated with five separate community nursing providers. More than a quarter of iGAS case episodes (25/80 [31.3%, Confidence Interval (CI) [21.3–42.6%]; 20/75 [26.7%, CI 17.1–38.1%] excluding the five cases with exposure >7 days prior to onset) in persons with preceding CHSDH care were associated with the five CHSDH associated outbreaks. Outbreaks had the following *emm* types: (*emm* 76.0, one outbreak of 11 episodes, including three recurrent episodes); and four further outbreaks, each comprising between two to four non-recurrent episodes of *emm* 89.0, 22.0, 82.1, and 1.3/1.0 (two of each emm type during the same time period associated with a single community nursing service). Three CHSDH outbreaks were limited to domestic households, and two occurred across both care home and domestic household settings.

All CHSDH-associated iGAS outbreak cases had closely related SNP relationships from their counterpart type-specific outbreak cases (maximum SNP distance between cases seven SNPs for the *emm* 76.0 outbreak, all cases were within 0–3 SNPs from another non-recurrent *emm* 76.0 outbreak case; for the remaining outbreaks all cases were within two SNPs or fewer of constituent cases). There were also three CHSDH-associated iGAS case episodes temporally associated with two community nursing services during outbreak investigations, but which did not meet the outbreak definition due to different *emm* type. Staff swabbing was undertaken in four outbreaks but did not identify a GAS colonization or infection. No environmental swabbing was conducted. Systematic swabbing of patients with wounds in one outbreak (*emm* 1.0) identified a non-invasive case within two SNPs of the invasive isolates.

On univariate analysis, there was evidence that outbreak episodes were associated with *emm* pattern E compared to A–C patterns (OR 6.1, 95% CI 1.8–20.9, *n* = 66), and the presence of skin or soft tissue infections (SSTI) in contrast to any other clinical presentations without co-occurring SSTI, such as iGAS pneumonia (OR 3.6, 95% CI 1.1–12.0, *n* = 79). We were unable to calculate ORs for the following variables with zero events in either the outbreak (*emm* pattern D; non-community nursing healthcare provider) or non-outbreak cases (recurrent episodes). The odds ratio for *emm* pattern genotype was robust to sensitivity analysis excluding three recurrent case episodes (which may have been consistent with the failure of eradication rather than reinfection) (OR 5.21, 95% CI 1.5–18.1, *n* = 63), but there was now weaker statistical evidence of disproportionate SSTI presentation (OR 3.1, 95% CI 0.92–10.4, *n* = 76).

## Discussion

In this cohort of iGAS cases associated with CHSDH, cases were elderly and had primarily received wound or ulcer care from community nursing services, findings which are consistent with the limited previous outbreak-based literature [5]. As in previous CHSDH outbreaks, investigations did not identify specific transmission routes [5], therefore we cannot definitively conclude that infection was healthcare acquired during CHSDH, and multiple potential infection sources or endogenous flora would need to be considered in community settings. However, our evidence suggests acquisition during CHSDH-healthcare is likely to have occurred in some cases, particularly within outbreaks, and may be a significant risk. Significant skin breakdown is a well-characterized risk factor for iGAS [1]; almost universal receipt of wound or ulcer care by cases (in 93.8%) would have increased vulnerability to infection and may be an important portal of infection entry. CHSDH was the most frequent shared exposure for all iGAS outbreaks (45%) during this period, and despite only 10% of adult cases noted as receiving CHSDH prior to onset, 31% of these CHSDH cases were linked to outbreaks. Three of the five outbreaks were also associated with concurrent invasive episodes of other *emm* types, which may be suggestive of wider infection prevention concerns [8]. OCTs did not identify other shared sources during the outbreak investigation, and our data showed cases infrequently reported symptomatic household contacts (8.8%) or other prior inpatient healthcare exposures (7.5%) which may have been plausible alternative sources of infection.

We observed high iGAS incidence and predominance of *emm* 1.0 strains in adults similar to national surveillance data during the 2022/2023 season (incidence 6.6/100000 [all ages], 19.0/100000 aged 75 and over; 53% of referred isolates in England *emm* 1.0 [all ages]) [11]. However, we found a much lower distribution of *emm* 1.0 infections in CHSDH-cases compared to other community cases, a finding not hereto reported. Similarly, we found that CHSDH outbreaks were associated with *emm* pattern E infections, A–C being under-represented (*emm* 1.0 being pattern A–C), in keeping with prior descriptions of CHSDH outbreaks where *emm* pattern E was more commonly observed [5]. This implies CHSDH-associated cases may have systematically different susceptibility, transmission modes, and/or sources of infection than other community cases. Assuming outbreak cases are more likely to be healthcare acquired, their association with *emm* pattern E suggests healthcare exposure may explain some of the variation in strain distribution between CHSDH cases and other community cases of the same age, in keeping with expected differences in contact patterns of relatively housebound CHSDH patients. *Emm* pattern is associated with tissue tropism, with pattern E potentially adapted to either throat or skin sites [10]. The pattern E strains in our study may have been more adapted to skin sites, leaving CHSDH patients with skin breakdown more vulnerable. The association of outbreaks with *emm* pattern E strains and with SSTI presentation (the association for the latter being weaker on sensitivity analysis) suggests tissue tropism and/or SSTI could also confer an increased transmission risk, perhaps during wound care and/or if cases with skin infection or colonization act as GAS reservoirs. However, the role of tissue tropism is uncertain, as some pattern E strains are adapted to throat infection/colonization, and there may be other traits associated with the outbreak strains that confer a transmission advantage in this population. Detection bias may also play a role, as *emm* pattern E strains were relatively uncommon among wider iGAS cases nationally [11], and uncommon strains may lead to easier cluster detection and variable assessment of CHSDH exposure. Our results may also be skewed by a single large outbreak (*emm* 76.0 is pattern E).

CHSDH-associated iGAS case fatality rate, defined as the percentage of all-cause deaths within 30 days of iGAS onset in this cohort, was 26.3%. This high mortality rate is in keeping with the 26% observed for cases aged 75 and over in 2022/2023 [11], and 29% in the 2021 PHE CHSDH report [5], although not directly comparable due to the variation in the length of follow-up used to define mortality outcome measurement end-point. Future study should use harmonized definitions of all-cause mortality, consider cause-specific mortality, and assess risk factors for mortality in CHSDH cases and outbreaks.

Using routine data and case or informant recall during contact tracing may result in misclassification of CHSDH exposure, mostly likely erroneously low, which may result in over-estimation of the percentage associated with outbreaks (due to too small a denominator), but may also impede outbreak detection, leading to potential under-estimation of the cases associated with outbreaks. In clonal strains with few accrued mutations genomic clustering alone may not reliably discriminate between epidemiologically un-linked cases, erroneously over-estimating outbreak cases. However, SNP differences were small, in keeping with previous shared exposure source outbreaks (0–7 SNPs) [8], and OCTs’ use of combined epidemiologic and molecular criteria to define outbreaks makes significant over-estimation much less likely. Conversely, it is likely we missed some CHSDH-associated outbreaks or outbreak cases, under-estimating the associated percentage of cases: non-invasive GAS infection is not notifiable so we may not detect outbreaks with multiple GAS cases and only a single iGAS case, and small iGAS outbreaks of more common *emm* types can be hard to detect in geographically dispersed cases [5] as WGS SNP analysis is requested on suspicion rather than being routine for all isolates [2]. Population-based approaches to GAS infection typing and sequencing, and the use of additional data sources and linkage to better identify shared exposures may provide stronger evidence on transmission routes.

These preliminary findings should be confirmed and developed with larger and more accurate and detailed datasets and analyses, ideally supported with wider use of sequencing and enhancements to surveillance data collected whilst investigating CHSDH-associated cases. Our analysis, consistent with previous evidence from iGAS outbreaks linked to wound care, suggests that CHSDH is associated with an elevated risk of propagation of iGAS outbreaks, and thus a significant number of cases may be preventable. High case fatality, and the potential for large outbreaks, demonstrate the possible serious clinical and public health consequences following GAS infection in this context and the importance of community services being supported to adhere to wound management and infection prevention guidelines. Additionally, whilst HPTs already implement recommended control measures on outbreak recognition [2, 6, 11] any delay may allow propagation and lead to larger and more complex outbreaks. Therefore, in line with iGAS healthcare and care home guidelines [2, 6], epidemiological investigation and review of infection control procedures should follow even a single case. These findings also underline the need for careful assessment of healthcare prior to iGAS onset, and rigorous outbreak surveillance, including systematic use of molecular typing approaches. Future iterations of iGAS healthcare-associated infection guidelines should consider including specific recommendations for cases with exposure to CHSDH wound and ulcer care.

## Data Availability

Any queries regarding the data can be directed to the authors who will consider data requests within the UKHSA data release policy.
